# Short-term outcomes and costs analysis of robotic-assisted versus laparoscopic cholecystectomy—a retrospective single-center analysis

**DOI:** 10.1007/s00423-023-03037-6

**Published:** 2023-08-08

**Authors:** Antonia Gantschnigg, Oliver Owen Koch, Franz Singhartinger, Peter Tschann, Wolfgang Hitzl, Klaus Emmanuel, Jaroslav Presl

**Affiliations:** 1 Department of General, Visceral and Thoracic Surgery, Paracelsus Medical University/ Salzburger Landeskliniken (SALK), Salzburg, Austria; 2 Department of General and Thoracic Surgery, Academic Teaching Hospital, Feldkirch, Austria; 3 Department of Ophthalmology and Optometry, Paracelsus Medical University/ Salzburger Landeskliniken (SALK), Salzburg, Austria; 4 Research Program Experimental Ophthalmology and Glaucoma Research, Paracelsus Medical University, Salzburg, Austria; 5 Department of Research and Innovation, Paracelsus Medical University, Salzburg, Austria

**Keywords:** Robotic-assisted cholecystectomy, Laparoscopic cholecystectomy, Operation time, Costs

## Abstract

**Purpose:**

Robotic-assisted surgery is an alternative technique for patients undergoing minimal invasive cholecystectomy (CHE). The aim of this study is to compare the outcomes and costs of laparoscopic versus robotic CHE, previously described as the major disadvantage of the robotic system, in a single Austrian tertiary center.

**Methods:**

A retrospective single-center analysis was carried out of all patients who underwent an elective minimally invasive cholecystectomy between January 2010 and August 2020 at our tertiary referral institution. Patients were divided into two groups: robotic-assisted CHE (RC) and laparoscopic CHE (LC) and compared according to demographic data, short-term postoperative outcomes and costs.

**Results:**

In the study period, 2088 elective minimal invasive cholecystectomies were performed. Of these, 220 patients met the inclusion criteria and were analyzed. One hundred ten (50%) patients underwent LC, and 110 patients RC. There was no significant difference in the mean operation time between both groups (RC: 60.2 min vs LC: 62.0 min; *p* = 0.58). Postoperative length of stay was the same in both groups (RC: 2.65 days vs LC: 2.65 days, *p* = 1). Overall hospital costs were slightly higher in the robotic group with a total of €2088 for RC versus €1726 for LC.

**Conclusions:**

Robotic-assisted cholecystectomy is a safe and feasible alternative to laparoscopic cholecystectomy. Since there are no significant clinical and cost differences between the two procedures, RC is a justified operation for training the whole operation team in handling the system as a first step procedure. Prospective randomized trials are necessary to confirm these conclusions.

## Introduction

Minimal invasive surgery has gained acceptance and popularity over the past decades in many visceral surgical procedures [1–4]. Laparoscopic cholecystectomy (LC) is one of the most frequently performed abdominal surgical procedure worldwide [5]. Since its introduction in 1987, LC has become the standard of care for symptomatic cholecystolithiasis as well as acute or chronic cholecystitis, as it has been shown to be superior to the open approach with shorter patient convalescence due to less trauma and shorter hospital length of stay (LOS) [6–9].

Robotic-assisted surgery is undoubtedly a revolutionary, rapidly growing area in minimally invasive surgery, especially since the approval of the DaVinci™ System (Intuitive Surgical®, Sunnyvale CA) by the FDA (U.S Food and Drug Administration) in 2000 [10]. The robotic system enables minimally invasive procedures in a precession that can overcome the limitations of laparoscopy due to various technical advantages [11–14]. Especially in urology and gynecology, the number of robotic-assisted operations is steadily increasing [15, 16]. In contrast, the adoption appears to be slower in general surgery. However, technically demanding operations such as rectum resection, esophagectomy and gastrectomy, have proven safe and feasible, and the robotic surgical system might be able to bring groundbreaking advantages for the patients post-operative outcome [17–19].

Individual comparative publications have described robotic-assisted cholecystectomy (RC) as a safe and feasible surgical procedure, which, however, is associated with longer operating times and higher costs and therefore does not justify the use of this technology [20–23]. On the other hand, robotic-assisted cholecystectomy has been described to be of value in particular in the context of training surgeons in handling the robot system with a steep learning curve [24, 25].

Our aim was to compare robotic-assisted cholecystectomy with laparoscopic cholecystectomy in terms of operating time, length of hospital stay and costs in a high-volume tertiary academic center.

## Methods

A retrospective single-center analysis of all patients who underwent a minimally invasive cholecystectomy between January 2010 and August 2020 was performed.

The study was approved by the local ethics committee (“Ethikkommission des Landes Salzburg”; protocol-number: 1227/2021) and data were retrieved from the prospectively maintained hospital database in accordance with ethical review guidelines.

To avoid a selection bias, and for better comparability between the two groups by establishing a homogenous patient population, only conventional multiport laparoscopic cholecystectomies (LC) as well as robotic assisted cholecystectomies (RC), performed by the same five experienced surgeons were included. The DaVinci Robotic X™ System (Intuitive Surgical®, Sunnyvale CA) was implemented in January 2018 in our tertiary academic center. As surgical residents primarily perform conventional laparoscopic but not robotic assisted cholecystectomies, we analyzed the LC group between January 2010 and April 2020 to access a similar number of interventions for all 5 surgeons in both groups.

All five surgeons, included in the study, were highly experienced in performing LC and had to perform at least 10 RC during the study’s observation period. These surgeons received expert training on the DaVinci Robotic X™ System prior to their first robotic-assisted cholecystectomy and were supervised by external proctors during their first surgeries. All RC, including the first operation performed by each surgeon, where included in the analysis.

Allocation of patients to both groups was done in a non-systematic way. Patients were able to decide whether they wish to undergo LC or RC and had the option of refusing the robotic surgery in order to undergo conventional laparoscopic surgery instead. This was in no case desired.

Demographic data collection included age, sex, BMI (body mass index, kg/m^2^), ASA score (American Society of Anesthesiologists) and history of prior abdominal operations [26].

**Fig. 1:**
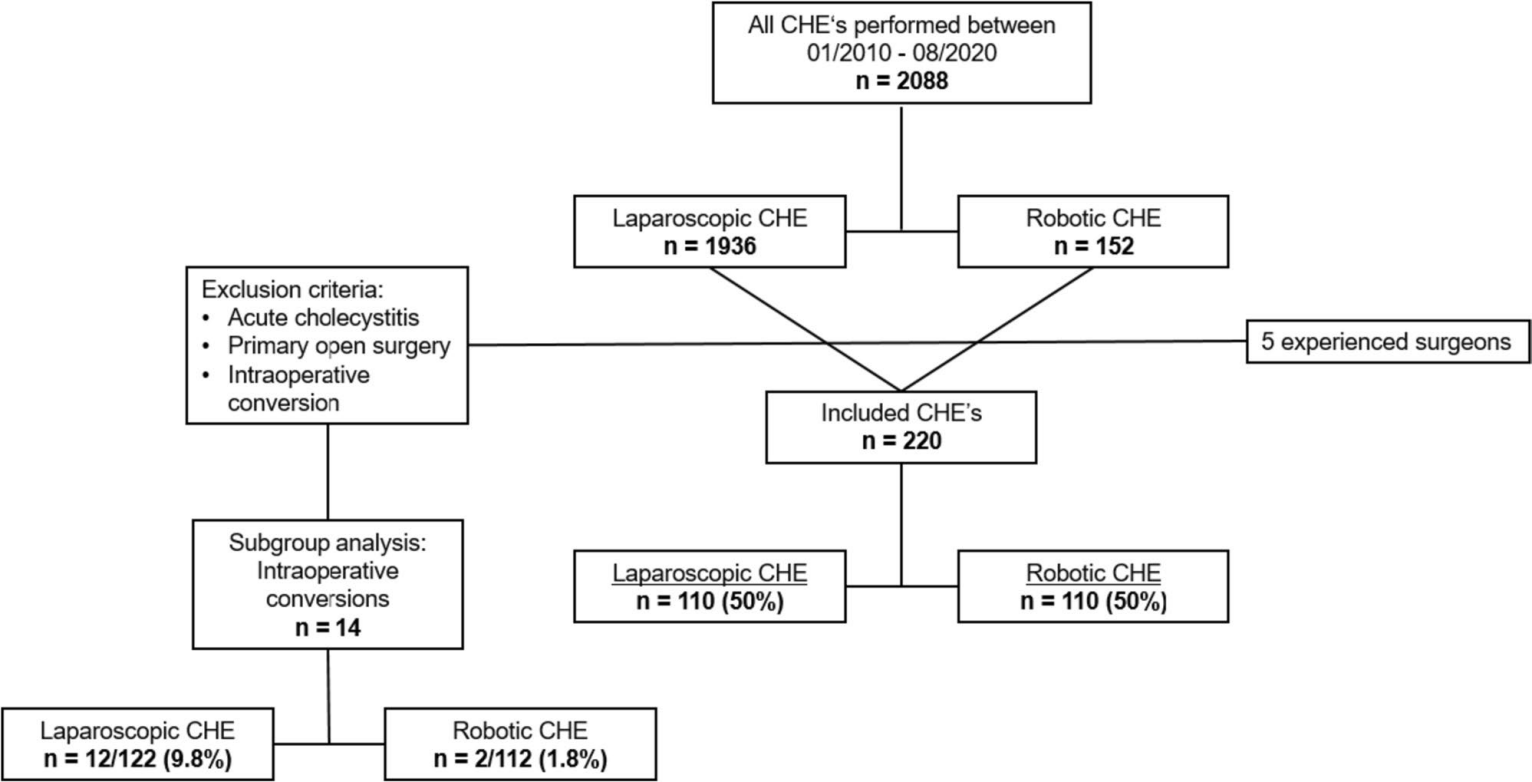
Flowchart of inclusion and exclusion criteria

Primary outcome measures were operation time, defined as incision to suture time, including robotic system set up with docking and undocking, postoperative length of stay, total LOS and intra-, and postoperative complications. Secondary outcome measures were demographic data. Inclusion criteria were age over 18, elective cholecystectomy due to symptomatic cholecystolithiasis or after biliary pancreatitis. Exclusion criteria were acute cholecystitis or primarily an open surgical technique (Fig. 1). Intraoperative conversion was analyzed secondarily in a subgroup analyze and is listed separately.

Cost evaluation was performed for laparoscopic and robotic cholecystectomy with evaluation of total costs per procedure including material costs, costs per mean operation duration and per mean hospital stay as well as hospital staff costs per day and per minute. Acquisition and maintenance costs were evaluated for both systems as well.

### Surgical technique

The same standardized surgical technique was used in all patients [27].

**Fig. 2:**
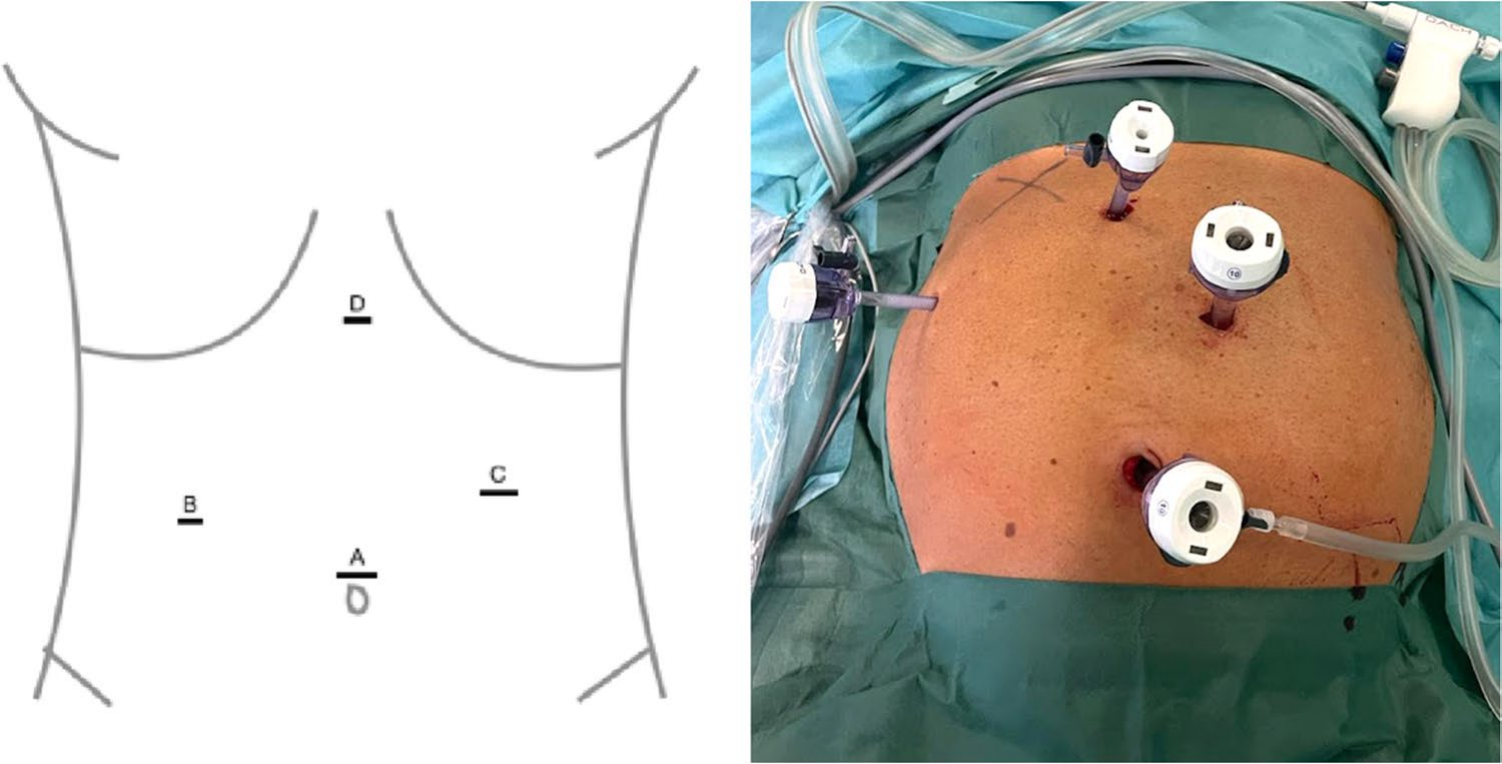
Port placement for laparoscopic cholecystectomy. A: 10-mm camera trocar, B: 5-mm surgeons left-hand trocar, C: 10-mm surgeons right-hand trocar, D: 5-mm assistant trocar

Conventional laparoscopic cholecystectomy was performed in French position with patient in reverse Trendelenburg left lateral position under general anesthesia. Port placement is displayed in Fig. 2. A 10-mm trocar was placed at the umbilicus for a 10-mm 30° optic camera (A). Another 10-mm trocar was placed at the left mid abdomen (C) and a 5-mm trocar at the right mid abdomen (B) for the surgeon’s instruments. An additional 5-mm trocar was placed at the xiphoid (D) for the assistant to retract the fundus of the gallbladder cranially.

**Fig. 3:**
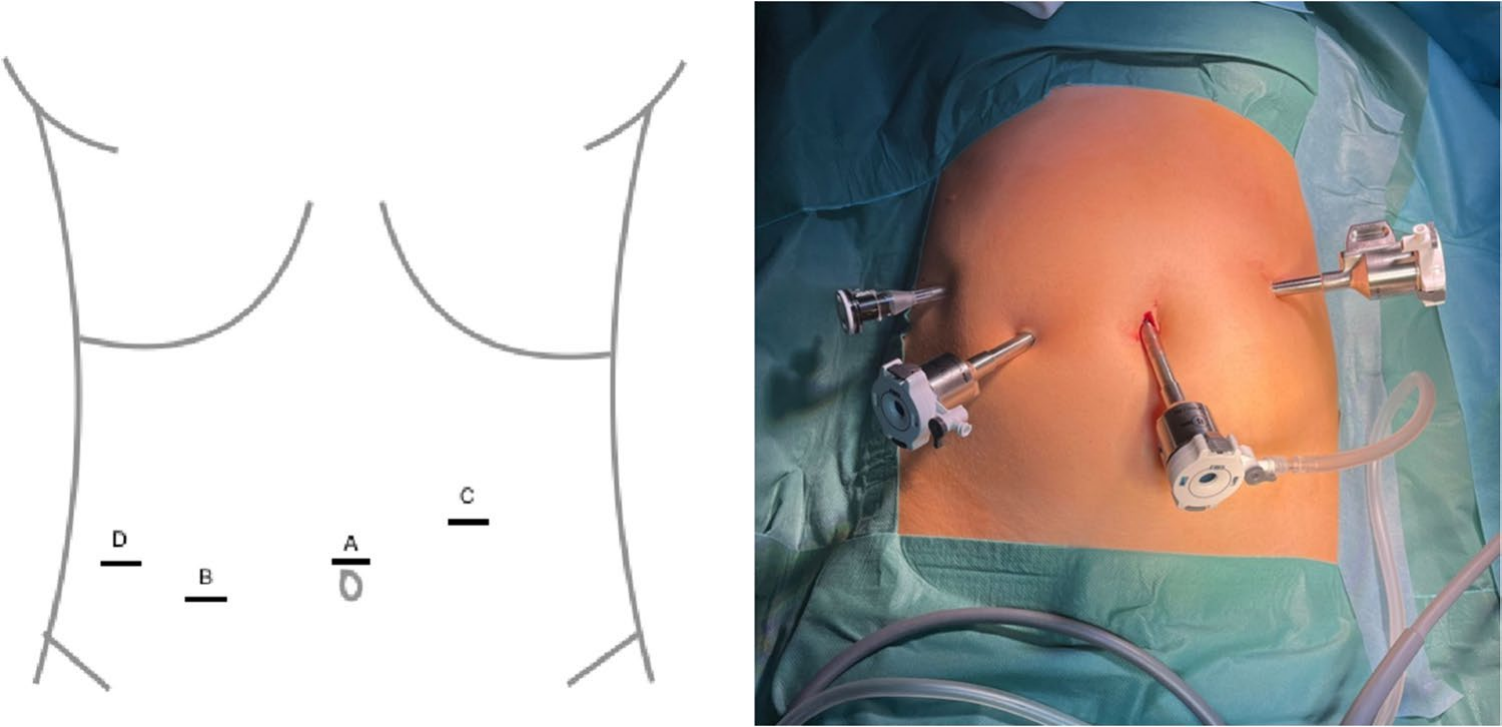
Port placement for robotic-assisted cholecystectomy. A: 8-mm camera trocar, B: 8-mm surgeons left-hand trocar, C: 8-mm surgeons right-hand trocar, D: 5-mm assistant trocar

Robotic-assisted cholecystectomy was performed with DaVinci Robotic X™ System (Intuitive Surgical®, Sunnyvale CA) in supine position with the same technique as the LC, expect for the port placement (Fig. 3). Here, 3 8-mm trocars were placed for the robotic arms (A, C and B) with an additional 5-mm trocar (D), around 6 cm lateral from trocar at position B, for the table assistant to retract the fundus of the gallbladder cranially.

### Statistics

Data were checked for consistency and normality. Fisher’s Exact test were used to analyze cross tabulations. Generalized linear model with log normal distribution, median tests and independent Student t-tests were used. All reported tests were two-sided, and *p*-values < 0.05 were considered statistically significant. All statistical analyses in this report were performed by use of STATISTICA 13 (Hill, T. & Lewicki, P. Statistics: Methods and Applications. StatSoft, Tulsa, OK).

## Results

In the study period 2088 elective minimal invasive cholecystectomies were performed. Of these, 220 patients met the inclusion criteria and were analyzed. 110 (50%) patients underwent LC, and 110 (50%) patients underwent RC by five experienced surgeons (Fig. 1).

Demographic data in both groups were comparable in terms of sex, age and BMI. ASA score distribution was comparable without significant differences as well. Patients’ characteristics are given in Table 1.

**Table 1:** Patients’ characteristics

Variables	RC (n = 110)	LC (n = 110)	p value
Mean age (y)	51.91 (SD ± 14.90)	55.05 (SD ± 18.02)	0.17
Sex n (%)			0.85
Female	68 (61.8)	67 (60.9)	
Male	42 (38.2)	43 (39.1)	
BMI (kg/m^2^)	28.11 (SD ± 4.79)	27.96 (SD ± 5.47)	0.82
ASA Score (%)			0.55
1	33 (30.0)	33 (30.0)	
2	67 (60.9)	62 (56.4)	
3	10 (9.1)	15 (13.6)	
Indication			0.45
Symptomatic cholecystolithiasis (%)	83 (80.9)	78 (70.9)	
After ERCP due to biliary pancreatitis (%)	27 (24.6)	32 (29.1)	
History of abdominal operation (%)	21 (19.1)	25 (22.7)	0.62
Operation duration (min)	60.2 (SD ± 15.4)	62.0 (SD ± 31.1)	0.58
Postoperative length of stay (days)	2.65 (SD ± 2.06)	2.65 (SD ± 1.52)	1.00
Conversion rate (%)	2/112 (1.79)	12/122 (9.84)	0.01*
History of abdominal operation (%)	1/2 (50.0)	7/12 (58.3)	

*Subgroup analysis of intraoperative conversions

There was no significant difference in the mean operation time between both groups (RC: 60.2 min vs LC: 62.0 min; p = 0.58). Median postoperative length of stay was similar in both groups (RC: 2.65 days vs LC: 2.65 days, *p* = 1).

No major organ injuries or bile duct injuries occurred in either group. In each group, one patient developed a postoperative wound infection without the need of further surgical interventions. In both the LC group and the RC group, relaparoscopy was required in one patient on the same day of the primary operation due to postoperative bleeding. In the laparoscopic operated patient, bleeding was found in the area of the umbilicus, in the robotic-assisted patient, bleeding was found in the liver bed. Both bleedings were stopped by electrocautery and no further complications occurred. One patient in the RC group developed an abscess in the right liver lobe, which resolved after CT-targeted drainage. One patient in the RC group developed postoperative urinary retention, which was treated with an intermittent catheter.

The distribution of indications for surgery was similar in both groups (*p* = 0.45). For both RC and LC, the indication for minimal invasive cholecystectomy was comparable with 83% vs 78% due to symptomatic cholecystolithiasis. All other patients, 27% in the RC group and 32% in the LC group, there was an indication for elective cholecystectomy after ERCP (endoscopic retrograde cholangiopancreatography) due to biliary pancreatitis due to choledocholithiasis in the context of cholecystolithiasis. History of prior abdominal operation was comparable between both groups as well with 19.1% in the RC group and 22.7% in the LC group (*p* = 0.62).

During the observation period of the study, in 14 patients a conversion from minimally invasive to open surgery took place, 12 times in the LC and 2 times in the RC group. In the LC group, the decision to convert to open operation was made due to extensive adhesions due to previous abdominal operations in 7 patients, complex and unclear anatomy in one case, bleeding in twos cases, and in two patients due to an injury of the small bowel during first trocar insertion.

In the RC group, reasons for conversion was once hemorrhage in the liver bed and once an extensive adhesion respectively.

An overview of the current overall charge, including material used for the individual operation, costs per mean operation duration as well as mean hospital stay, revealed slightly higher total median costs per operation and postoperative hospital stay for the robotic approach (€2088 vs €1726). Hospital stay per day and staff costs per minute are the same in both groups (Table 2). In Table 3 acquisition and maintenance cost of the robotic and laparoscopic operation system, respectively, was performed. The costs were calculated on the basis of our institute's internal calculation bases with a net service life of 10 years and 1000 hours per year (50% of the gross calculation with 250 days per year, 8 hours per day) for both the laparoscopic tower system and the robotic system. With a purchase price of over 2 million Euros, the DaVinci Robotic X™ System is significantly more expensive than a laparoscopy tower system.

**Table 2:** Cost statement RC and LC per procedure

Costs in € per procedure	RC	LC
Total Costs €	2087.69	1725.64
Material for surgery	810.31	437.0
Laparoscopic instruments and needle holders (resterilizable)	40.0	40.0
Laparotomy Cover Set (Legs Spread)	23.8	23.8
Da Vinci Xi Arm Drape	224.0	
Da Vinci XI Cautery Hook	180.0	
Da Vinci XI Fenestrated Bipolar Forceps	170.0	
Trocar set da Vinci (4 pieces)	16.0	
Laparoscopy 5 mm trocar	37.08	
Laparoscopic retrieval bag 5 mm	49.43	
Trocar set optical 5 mm (without blade)		60.42
Trocar set optical 5 - 11 mm (without blade)		47.40
Suction/flushing system	44.0	44.00
Laparoscopic retrieval bag standard		47.58
Disposable Tips L Hook		25.08
Hospital Endoscopy set		11.49
Laparoscopic scissors insert		62.72
HEM-O-LOK Polymer Clip Large (6 pieces per pack)	26.0	26.0
Costs per Mean Operation Duration	405.95	417.21
Costs per mean postoperative hospital stay	871.43	871.43
Hospital Stay Costs (per day)	328.84	328.84
Staff Costs (per Minute)	6.74	6.74

**Table 3:** Cost statement RC and LC acquisition and maintenance

Acquisition and maintenance costs in €	RC	LC
Calculated useful life (y)	10	10
Net hours per year (h)	1000	1000
Acquisition costs	2 015 918.0	114 205.0
Costs per use	276.0	13.0
Costs per year	201 592.0	11 420.0
Costs per hour	202.0	11.0
Costs per minute	3.36	0.19

The statement of costs does not include the costs of the data collective.

## Discussion

In this study, we could demonstrate robotic surgery as feasible and safe with a low conversion rate and expectable higher costs.

Robotic-assisted surgery is undoubtedly a revolutionary, rapidly growing area in minimally invasive surgery. The aim of this study was to compare the outcome and the costs of laparoscopic with robotic CHE in a single Austrian tertiary center.

In our retrospective single center analyze, both groups were comparable in terms of sex, BMI and ASA-score. Patients in the RC group were significantly younger. We have no clear explanation for this finding, but it is possibly reflecting the fact that younger patients are generally in better health with less comorbidities without prior abdominal operations, which could represent a certain selections bias.

No major organ or bile duct injuries occurred neither in the RC nor in the LC group. A significantly lower number of conversions were required in the RC group, mainly due to adhesions, with 1.8% (2 out of 112) compared to 9.8% (12 out of 122) in the LC group. Our findings are in accordance to the literature, also across different surgeries and specialties. In colorectal and gynecologic robotic surgery, a reduction of conversions to open surgery has been shown multiple times. Reasons could be, among other things, the completed learning curve at the start of the analysis, technical advantages of the robotic system and, selection bias in the selection of younger and fitter patients for the robotic approach [28–30].

Our results are in accordance with a meta-analysis from Huang et. Al, where 1,589 patients (laparoscopic cholecystectomy, *n* = 921; robotic cholecystectomy, *n* = 668) within thirteen studies, twelve retrospective trials and one randomized controlled trial were examined. They describe an intraoperative complications occurrence in a median of 0% in the both groups (range = 0–33.3 for LC; range = 0–41.7 for RC). Postoperative complications occurred in a median of 1.9% and 2.6% in the LC and RC groups range = 0–0 for LC, range = 0–33.3 for LC). The median conversion rate was 0% in both groups (range = 0–15.7 for LC, range = 0–1.9 for RC) [31].

Our results show a higher conversion rate (*p* = 0.01) in the LC group in comparison to RC. As Huang et al hypothesized this may be indicated due to a technologic advantage of the robot in more challenging cases where better view through 3-dimensional view, increased degrees of maneuverability of the instruments, and decreased physical stress on the surgeon may be able to prevent the need for operative conversion. Otherwise, a selection bias may be the cause. Surgeons seem to predominately choose the more established operative technique (LC) for more difficult situations, such as adhesions.

In our cohort, there was no difference in the number of patients with prior abdominal surgery (*p* = 0.62) or preoperative ERCP for choledocholithiasis and consecutive biliary pancreatitis. The fact that our cohort had a higher conversion rate in the LC group mostly due to adhesions, while there was statistically no difference in prior abdominal operations, strengthens the hypotheses that the robotic system might bring an operational advantage in difficult cases.

Postoperative and total hospital length of stay was similar in both groups, with a slight trend towards a hospital stay length reduction in robotic group which reflects the data in the literature [21]. This is an important factor, which might cause a cost reduction and should be considered in the future. However, further randomized trials are needed to proof the non-significant trend in this study.

The operation time (incision to closure time), including robotic system set up with docking and undocking was similar to the laparoscopic operation (*p* = 0.58; Table 1). In literature, the comparison of operating times is a highly discussed point, since the expenditure of time is one of the main disadvantages of the robotic system in different operations. While a meta-analysis from Han et. al identified longer operation times with the robotic approach [32], among others, Breitenstein et. al [20], and Ayloo et. al [21], both described similar times between RC and LC.

In our facility, the entire operation team, from the surgeons, scrub nurses, surgical techs to the anesthesiologists, has received extensive training in handling of the DaVinci Robotic X™ system and are routinely exposed to the robotic platform. This standardized the process and reduced docking times to a minimum.

Interestingly, Ayloo et. al also showed in their study, that operation times were similar in the RC group between young and experienced surgeons (53 vs 56 min) in contrast to 71 vs 40 min in the LC between young and experienced surgeons, underlying the hypothesis, that the younger generation of surgeons may need a shorter learning phase [21]. This can also be taken into account when arguing that robotic cholecystectomy would be a good training procedure while becoming accustomed to the surgical robot.

The robotic approach will probably not completely replace the laparoscopic approach for cholecystectomy in the future. However, it still finds its justification with regard to the training of the surgeons and the whole operation team in handling the system as a first step procedure before more complex once in academic tertiary centers with access to robotic surgical systems [10, 31, 33].

Many studies have noted significantly higher costs associated with the use of robotics for minimally invasive cholecystectomies in the past and have not concluded any justification for their use until the price is reduced [20, 31, 34–36]. The most significant cost difference lies the initial investment for the purchase the robotic system with just over 2 million Euros. In our institution the costs per operation between the robotic and laparoscopic approach are comparable which is caused not only by a permanent reduction of the robotic instruments prices but also due to the increased use period of the instruments. Hereby, a price approximation was achieved. Nonetheless, the robotic approach is still slightly more expensive due to higher material costs.

It should be noted that this study has few limitations: First, this study was designed retrospectively. Therefore, the evaluation period for the LC is longer and further in the past than for the RC group, which might have an influence on the total length of hospital stay and thus, the costs per median hospital stay. Second, only operations performed by five experienced surgeons after completed learning curve in laparoscopic cholecystectomy were included. The learning curve of the robotic approach was included in the observational period of our study, as all RC performed by each surgeon, including their first operation, was included. Then again, although the learning curve is included in the RC group the operation duration did not differ during the observational period, which might be due to the fact, that all surgeons included are highly experienced. As we have mentioned that the justification for the robotic approach is particularly evident in the surgeon training process, a study comparing the outcomes of inexperienced surgeons would be of interest. Third, the potential selection bias of the decision on the operation technique for patients after ERCP in the same hospital stay towards conventional laparoscopy could be the reason for a longer total length of hospital stay in the LC group as these patients have a prolonged hospital stay preoperatively. In addition, the observational period in the LC group was significantly longer than in the RC group, which could also be part of a longer hospital stay. For these reasons, we describe postoperative hospitalization comparing the two techniques according to postoperative mortality rather than total length of hospitalization. Fourth, the cost analysis was performed using the actual cost of materials, staff and hospital stay per day and does not take into account price evolution over time.

In order to obtain more comparable results, randomized controlled trials including an in-depth cost analysis are necessary.

## Conclusion

Our data shows, in concordance with previous literature, that robotic-assisted cholecystectomy is comparable to laparoscopic cholecystectomy in terms of patient safety, operation time and postoperative hospital length of stay [20–22]. We observed is significantly lower conversion rate in the robotic group. To date, robotic-assisted cholecystectomy can be performed without a significant cost difference excluding acquisition costs for the robotic operation system.

## Abbreviations

CHE: Cholecystectomy
ERCP: Endoscopic retrograde cholangiopancreatography
LC: Laparoscopic cholecystectomy
LOS: Length of stay
RC: Robotic-assisted cholecystectomy
